# Design and baseline data of a population-based metabonomics study of eye diseases in eastern China: the Yueqing Ocular Diseases Investigation

**DOI:** 10.1186/s40662-019-0170-1

**Published:** 2020-01-19

**Authors:** Yuxuan Deng, Yuanbo Liang, Sigeng Lin, Liang Wen, Jin Li, Yue Zhou, Meixiao Shen, Jingwei Zheng, Kemi Feng, Yanting Sun, Kwapong Willaim Robert, Jia Qu, Fan Lu

**Affiliations:** 1 Clinical and Epidemiological Research Center, Eye Hospital of Wenzhou Medical University, 270 Xueyuan Road, Wenzhou, 325027 Zhejiang China; 2 School of Ophthalmology and Optometry, Wenzhou Medical University, 270 Xueyuan Road, Wenzhou, 325027 Zhejiang China; 3 Qingdao Municipal Hospital, 5 Donghai Middle Road, Qingdao, 266071 Shandong China; 4 Wuhu First People’s Hospital, 1 Chizhushandong Road, Wuhu, 241000 Anhui China; 5 Eye Hospital of Fushun City, 1 Hupo Road, Fushun, 113006 Liaoning China; 6 Qilu Hospital of Shandong University (Qingdao), 758 Hefei Road, Qingdao, 266035 Shandong China

**Keywords:** Population, Baseline, Ophthalmic epidemiology, Visual impairment, Screening, Metabonomics

## Abstract

**Background:**

China is undergoing a massive transition toward an urban and industrial economy. These changes will restructure the demographics and economy which will eventually influence the future patterns of disease. The risk factors of vision-impairing eye diseases remain ambiguous and poorly understood. Metabolomics is an ideal tool to understand and shed light on the ocular disease mechanisms for earlier treatment. This article aims to describe the design, methodology and baseline data of the Yueqing Ocular Diseases Investigation (YODI), a developed county population-based study to determine the prevalence and primary causes of visual impairment; also with metabonomics analysis we aimed to identify, predict and suggest some preventive biomarkers that cause blindness.

**Methods:**

A population-based, cross-sectional study. Randomized clustering sampling was used to identify adults aged 50 years and older in Xiangyang Town, Yueqing county-level City. The interviews covered demographic, behavioral, ocular risk factors and mental health state. The ocular examination included visual acuity, autorefraction, intraocular pressure, anterior and posterior segment examinations, fundus photography, retinal tomography and angiography, and visual field testing. Anthropometric measurements included height and weight, waist and hip circumference, blood pressure, pulse rate, electrocardiogram, and abdominal ultrasound scan. A venous blood sample was collected for laboratory tests and metabonomics studies.

**Results:**

Of the 5319 individuals recruited for the YODI, 4769 (89.7%) subjects were enrolled for analyses. The median age was 62.0 years, and 45.6% were male. The educational level of illiteracy or semi-illiteracy, primary, middle and high school or above was 29.8%, 45.5%, 20.1%, and 3.3%, respectively. Majority of the participants were female, younger, and less educated when compared with nonparticipants. The average body mass index and waist-hip ratios were 24.4 ± 3.4 kg/m^2^ and 0.9 ± 0.1 respectively. Blood sample collection reached a sample size of 1909 (479 from subjects with self-reported diabetes and 1430 from one-third of the 4290 subjects without self-reported diabetes).

**Conclusions:**

The YODI provides population-based data with a high response rate (89.7%) on the prevalence and primary causes of major vision-impairing eye diseases in developed county areas in eastern China. Metabonomics analysis from YODI will provide further association of metabolic characteristics with the visual impairment eye diseases. The risk prediction model could be created and has the potential to be generalized to developed eastern areas in China for prevention.

## Background

In the last three decades, there have been a series of population-based surveys on eye studies worldwide (the United States [1–6], Western Europe [7–11], Australia [12–15], Singapore [16–23], Japan [24–27], and China [28–33]). These studies have served as valuable guidelines for primary eye healthcare and prevention of blindness. However, visual impairment diseases are usually undetected during the early phase or genesis of the disease cascade until there’s a deterioration to the vision or detection of clinical signs. The risk factors of vision-impairing eye diseases such as diabetic retinopathy (DR) remain ambiguous and poorly understood. The predictive factors (glycosylated hemoglobin and duration of diabetes) only accounted for nearly 11% in the variation of DR risk in the Diabetes Control and Complications Trial [34, 35]. Due to the inconclusive and inconsistent reports from previous reports, challenges have arisen in identifying genetic risk factors. Studying the genetic intermediate associations is considered as an advanced route to improve our understanding of the complex ocular disorders [36].

The metabolites are regarded as close representatives of an immediate cellular state within a biological system, considering the genomic cumulative effects and interactions with lifestyle- and environment-related exposures [37]. Metabolomics, as a detailed measurement of the metabolome profile, is an ideal tool to understand and shed light on the disease mechanism in order to help in the earlier treatment of the disease [38]. Recent reports have demonstrated the good applicability and prospect of metabolomics for the study of ocular diseases [39–43]. Thus, our aim was to find novel metabolic biomarkers associated with the progression of vision impairment in a population for the earlier detection, diagnosis, and prognosis with a therapeutic target.

Chinese populations were the main research objective in some studies outside mainland China, such as Taiwan province [44], Hong Kong SAR [45], Singapore [46], and the United States [47]. China is currently undergoing a massive transition toward an urban and industrial economy; as such, county rural industrialization promotes local urbanization with the rise of township businesses. Consequently, these changes are restructuring demographics and economy. This big transition will have an influence on future patterns of disease. For example, the prevalence rate gap of chronic diseases was significantly reduced between Chinese urban and rural areas from 2008 to 2013 [48]. Thus, we also aim to provide estimates of the prevalence and risk factors of various ocular diseases in a large population as a representative model for other developed county areas in eastern China.

**Fig. 1:**
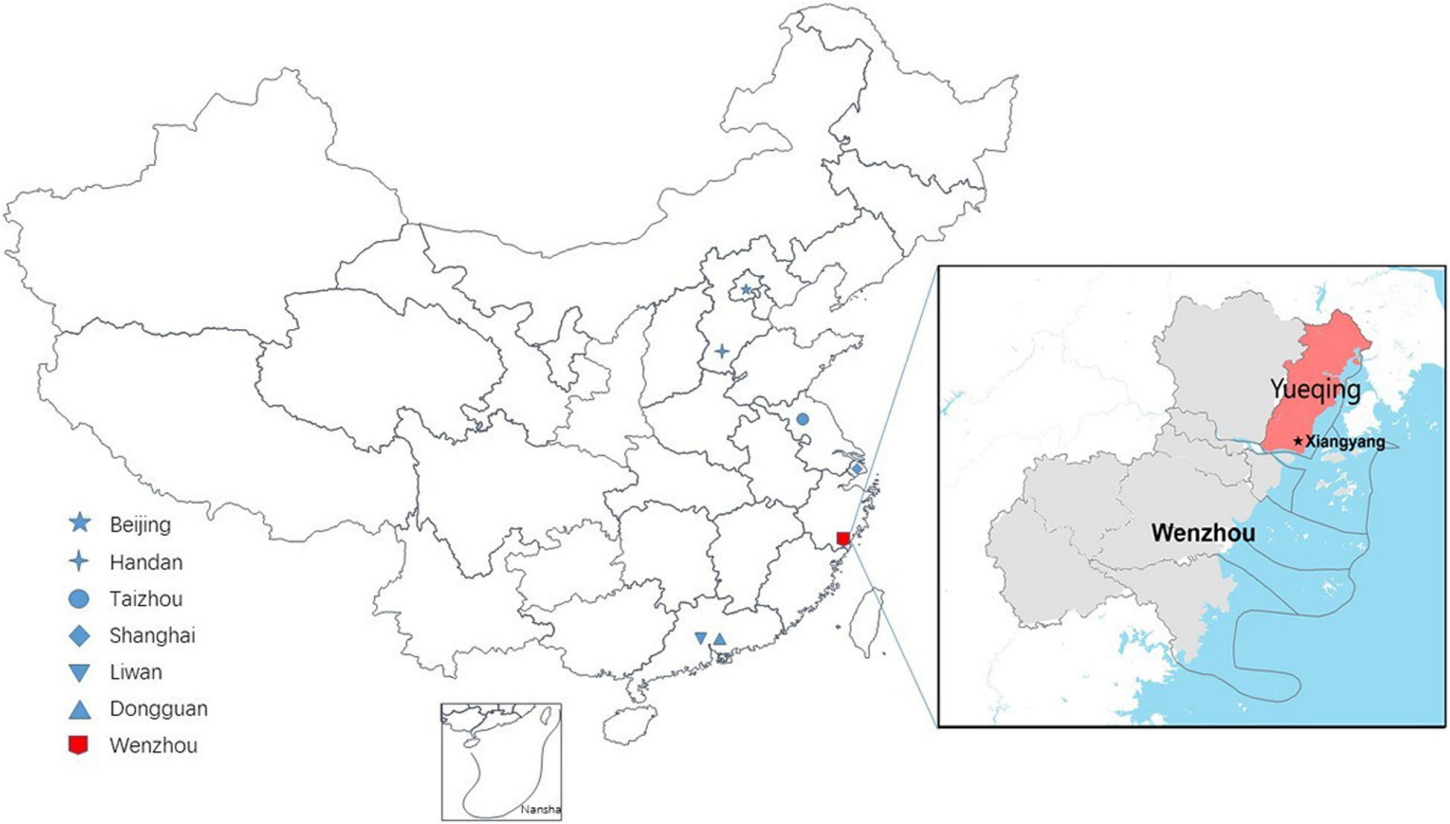
The location of Yueqing Eye Diseases Investigation and previous eye studies in eastern China

The top 100 counties in China (70 counties in eastern China) account for only 7% of the national population, but 10% national gross domestic product (GDP) and 25% GDP of all 1879 counties in 2019 [49, 50]. Yueqing county-level city, located in eastern China under the administration of Wenzhou City, Zhejiang Province (Fig. 1), is representative of the top counties (ranked 16th in 2019) [49]. In addition, Yueqing has higher employment in industry according to the 2010 National Census (Table 1) [51, 52]. Xiangyang Town, in south Yueqing, has a total area of 14.75 km^2^ with a jurisdiction of over 35 administrative villages. According to the demographic data from the local police station (using the Household Resident Register record kept by the local police station), there is a stable population of about 12,300 people aged 50 years and older out of the 39,900 registered residents. Xiangyang Town is thought to be one of eastern China’s typical models of economic development for a developed county. For example, in 2018, Xiangyang’s per capita gross disposable income was 5467 dollars (China rural areas: 2208 dollars; China urban areas: 5929 dollars; 1 dollar is equivalent to 6.62 yuan in 2018) [53, 54].

**Table 1:** Comparing the demographic characteristics of Yueqing with Chinese rural and urban areas according to the 6^th^ National Census taken in 2010

Characteristics	Yueqing county-level city	Rural areas	Urban areas
Population	1,389,332	670,005,546	662,805,323
Per capita disposable income ($)^a^	4494 (Urban area) 2038 (Rural area)	874	2823
Gender (%)			
Male	52.1	51.2	51.2
Female	47.9	48.8	48.8
Age (%)			
0–9	11.1	9.2	12.8
10–19	12.7	12.9	13.3
20–29	19.8	19.2	15.0
30–39	19.2	18.1	14.2
40–49	16.5	17.5	17.0
50–59	9.7	11.4	12.6
60–69	5.7	6.6	8.4
70–79	3.6	3.8	4.8
≥ 80	1.7	1.3	1.8
Education (%)^b^			
Illiteracy or semi-illiteracy	7.0	7.2	2.8
Primary school	30.8	38.0	19.8
Middle school	39.6	44.9	38.6
High school	15.4	7.7	22.1
College and above	7.2	2.1	16.7
Employment in three industries (%)			
Agriculture	8.7	74.8	16.0
Industry	60.6	15.9	34.3
Service	30.7	9.3	49.7
Minority (%)	2.7	11.2	5.5

^a^1 dollar is equivalent to 6.77 yuan in 2010

^b^“illiteracy” was defined as an inability to read any Chinese words; “semi-illiteracy” was defined as having some understanding of Chinese words, but obtained little to no useful information through reading

With support from the local government, we started the Yueqing Ocular Diseases Investigation (YODI) in Xiangyang Town to promote primary eye healthcare and prevention of visual impairment. This article presents the design and methodology in this study and summarizes the baseline data of this population.

## Methods

### Study design and specific aims

The YODI is an observational cross-sectional, population-based study on residents aged 50 years or older in Xiangyang Town of Yueqing. It was funded by the Science & Technology Department of Zhejiang Province and carried out from June 2018 to May 2019. This study adhered to the principles of the Declaration of Helsinki and ethics committee approval was obtained from the Eye Hospital of Wenzhou Medical University.

The YODI sought to attain three specific research objectives:

1. The prevalence and primary causes of visual impairment in Xiangyang Town (≥50 years).
2. The prevalence and risk factors of visual impairment diseases in Xiangyang Town (≥50 years).
3. To build a risk prediction model from blood metabonomics analysis of corresponding ocular diseases such as cataract, glaucoma, DR and age-related macular degeneration (AMD).

### Sampling and recruitment strategies

Based on the previous research [28, 32, 33, 55–61], we assumed the prevalence of the main ocular diseases to be 2% or above in this study. A sample of 4517 was estimated under a precision of 0.005, a confidence level of 95%, and a design effect of 1.5 [28]. Based on calculations, a sample size of 5904 subjects was sufficient considering 90% accuracy of registration information and an expected response rate of 85%.

A clustered sampling frame was used wherein one natural village or two or three lightly populated villages were regarded as one cluster to reach a similar size. Of the 35 villages in Xiangyang Town, we randomly selected 16 of them to obtain a target sample size of 5938. Using the Household Resident Register record provided by the local government, we derived the sampling frame from an official list of names after checking the names with each village’s doctor and a cadre. A brochure with an invitation card was sent to each resident on the sampling list. A door-to-door visit was also made to their homes by recruitment staff to confirm eligibility status on three workdays. Health lectures, free pickup services, and breakfast were used to improve the response rate. Residents aged 50 years or older were considered “eligible” if he/she had lived in the residing area for more than half a year and were living without mental or terminal illness. The eligible subject was then appointed to the clinic for an eye examination. Written informed consent was obtained from all subjects after explaining each step of the examination along with possible benefits and risks. The participants who could not read nor write were asked for informed consent with a handprint. Finally, a total of 5319 persons were confirmed eligible.

### Central clinical examination

**Fig. 2:**
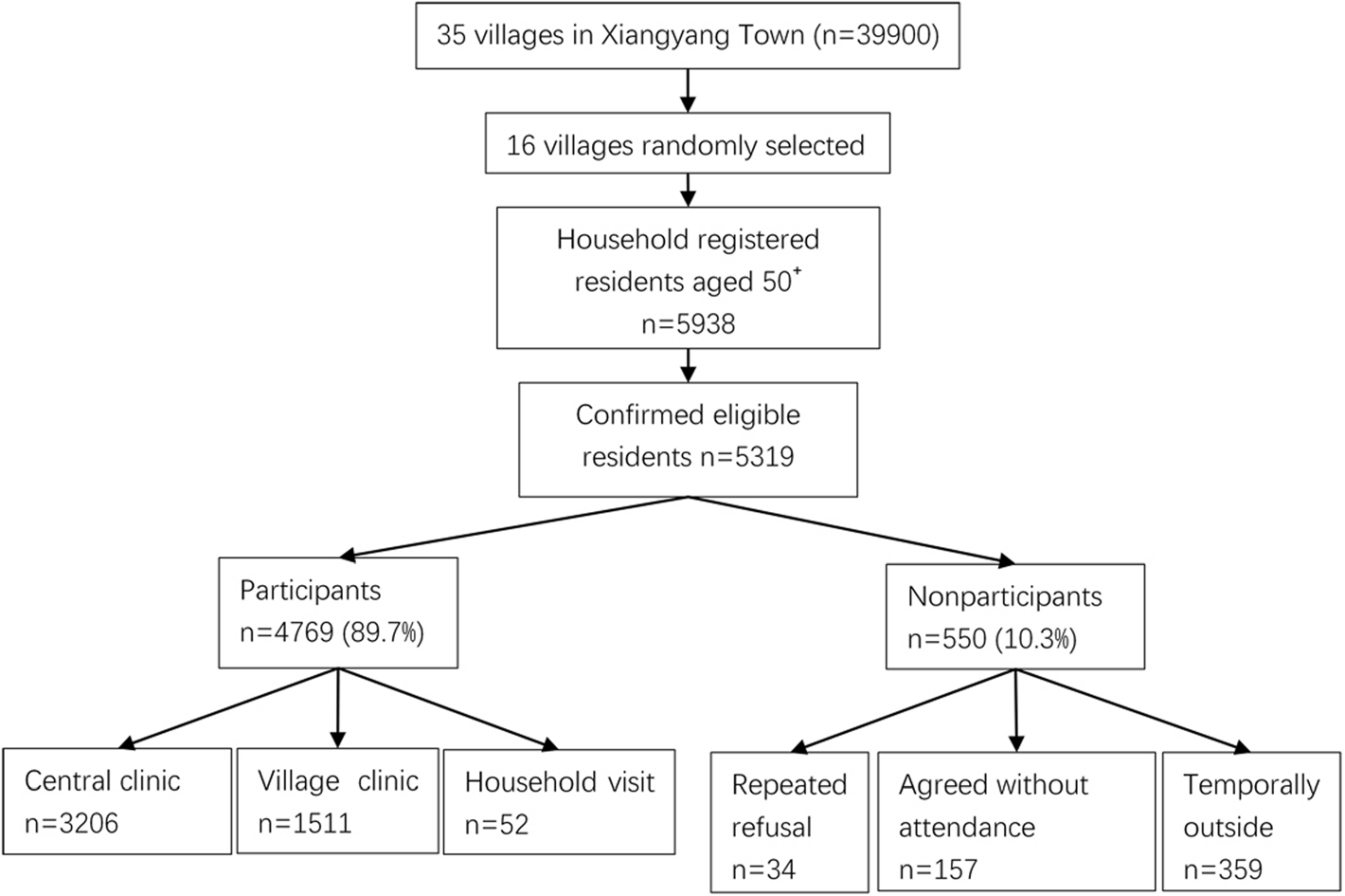
Flowchart for completing the target subject size and survey process

At the Xiangyang Health Center, a standardized examination item was conducted and summarized in Fig. 2.

1. Registration: The resident's eligibility was reconfirmed, and registration was done with their identity cards. The demographic details and written informed consent were obtained. Self-reported diabetes was confirmed and noted during registration before the blood draw.
2. Anthropometric examination: The height was measured with a wall-mounted measuring tape in centimeters. The weight was measured with a bathroom scale (RGZ-120, Suhong, Jiangsu, China) in kilograms. The hip and waist circumference was measured in centimeters. The operational process was based on methods described previously by Peng et al. [62].
3. Pulse rate and blood pressure: Participants sat at a table quietly with the back supported and with both feet flat on the floor for 5 min before blood pressure measurement on the right arm. Pulse rate and systolic and diastolic blood pressure (SBP & DBP) were recorded with an electronic automated blood pressure monitor (J30, OMRON, Matsusaka, Japan).
4. Autorefraction and Visual Acuity (VA): Presenting distance VA (PDVA) was measured monocularly (starting with the right eye) and binocularly with participants’ existing optical correction, using an International Standard VA Chart with a standard lightbox (XK100, Xingkang, Wenzhou, China) at a 5-m distance. Every optotype was given 3 s to read. If no optotypes were read, the participant was moved closer to the chart, and VA was calculated using the following formula: (0.1 × distance) / 5′ [63], allowing acuities as low as 0.02 at one meter. If any optotypes were still unidentifiable, VA was tested as counting fingers, hand movements, light perception, or no light perception. For those with PDVA worse than 0.5 (6/12) in either eye, the best-corrected VA (BCVA) was obtained using a trial frame to refine the autorefraction (ARK-1, NIDEK, Hiroishi, Japan) readings. The results were expressed with a Snellen equivalent form.
5. Intraocular Pressure (IOP): IOP was measured by a non-contact Tonometer (NT-510, NIDEK, Hiroishi, Japan). The final IOP was the average of three independent IOPs measured in each eye. Palpation estimation was used if IOP was unmeasurable for corneal abnormalities.
6. Slit-lamp Examination: The slit lamp examination (LS-5, Sunkingdom, Chongqing, China) for anterior and posterior segments were performed by ophthalmologists. The initial examination confirmed the anterior segment diseases (e.g., corneal abnormalities, pterygium) and the anterior chamber depth by the method of Van Herick [64]. The compound tropicamide eye drops (containing 0.5% tropicamide and 0.5% phenylephrine hydrochloride) were used and repeated to get the best possible mydriasis. Pupil dilation was performed for participants without IOP more than 21 mmHg, temporal corneal limbus depth grade < 25% of corneal thickness, and a history of glaucoma and coronary heart disease. Twenty min after the mydriasis, the supplemental examination was undertaken for grading of posterior segments and cataract using the Lens Opacities Classification System II (LOCS-II) as reference [65].
7. Visual field test: All glaucoma suspects were examined by perimetry using the Humphrey Visual Field Analyzer 750i (Carl Zeiss, Jena, Germany) in the 24–2 SITA static mode.
8. Fundus photography: Experienced photographers used a digital non-mydriatic fundus camera (CR-2 AF, Canon, Tokyo, Japan) to obtain bilateral retinal 45-degree images of the optic nerve (centered on the disc) and the macula (centered on the fovea) [66]. Fundus photographs were graded preliminarily for the level of DR and other fundus lesions by two graders. The kappa value calculated for intra-grader and inter-grader agreement on fundus photographs were 0.91 and 0.88, respectively.
9. Retinal tomography and angiography: Intraretinal and choroid structures were imaged by a Spectral Domain-Optical Coherence Tomography Angiography system (OCT-HS100, Canon, Tokyo, Japan) using the radial mode (fixation position: macula, 10 mm diameter; 12 lines). In addition, 3 × 3 mm macula and disc angiography were performed to obtain microvascular images of retinal capillary plexus around the macula. We used a custom algorithm to quantify the segmentation of retinal thickness and the superficial and deep macular microvascular network [67, 68].
10. Blood collection for biochemistry tests and metabonomics analysis: Using one sterile vacuum tube with and one without ethylene diamine tetraacetic acid (EDTA) to collect 1 mL venous blood of each kind of tube for biochemistry tests from all 4709 subjects. All participants with a history of diabetes (479/4709) and one of every three participants (1430/4230) without self-reported diabetes had additional 2mL of blood drawn for each tube collected. These 1909 subjects had 6 mL of blood collected in total of which 2 mL were for biochemistry tests and more 4 mL for additional testing. Participants were asked to fast for more than 8 h and fasting venous blood was collected between 7:00 and 8:00 a.m. On the same day, all biochemistry tests were undertaken by the laboratory at the Eye Hospital of Wenzhou Medical University. A 1 mL EDTA-tube of blood was analyzed for levels of glycosylated hemoglobin (HbA1c) and 1 mL serum was analyzed for levels of (a) fasting blood glucose (FBG), (b) blood urea nitrogen (BUN), (c) serum creatinine (Scr), and (d) lipids: total triglycerides (TC), total cholesterol (TG), high-density lipoprotein-cholesterol (HDL-C) and low-density lipoprotein-cholesterol (LDL-C). After centrifugation (1500 rpm, 10 min, 20 °C), nearly 1 mL serum and 1 mL plasma from each tube of 2 mL blood samples were aliquoted for 0.3 mL in three 1.5 mL vials with O-rings and stored at − 80 °C for future metabonomic analyses.
11. Questionnaires: The questionnaires were administered by trained interviewers during pupil dilatation. The questionnaires included diabetes and hypertension risk assessment questionnaire developed by the Dongguan eye study [33], the Mini-Mental State Examination (MMSE) for cognitive state [69], and the Geriatric Depression Scale (GDS) for persons older than 55 years old [70].
12. Other physical measurements: Abdominal ultrasound scan (Z6, Mindray, Shenzhen, China) and electrocardiogram measurement (FX-8322, Foton, Tokyo, Japan) were performed on all participants.

### Supplementary village examination

We conducted a supplementary exam in the selected village centers for eligible participants who did not attend the central clinical examination. This examination included:

1. Demographic data registration
2. Blood sample collection
3. Heart rate, blood pressure, height, weight, waist and hip circumference
4. PDVA
5. Non-contact IOP
6. Slit-lamp biomicroscopy
7. Questionnaires
8. Dilated fundus photography (field 1 and field 2)
9. Abdominal ultrasound scan and electrocardiogram measurement

The examination procedures above were consistent with those undertaken at the central clinic.

### Household examination

We performed a limited survey of residents who were unable to attend the village clinical examination. The questionnaires were collected after the demographic data registration. The physical examination included blood pressure, heart rate, waist circumference, and hip circumference. The ophthalmic examination included PDVA, hand-held slit-lamp microscopy (LS-1B, Sunkingdom, Chongqing, China) and direct ophthalmoscopy (BETA 200S, HEINE, Herrsching, Germany).

### Organizational structure

The organizers of the YODI engaged the conductors of the Handan Eye study [28] and Fushun diabetic retinopathy study [71] to supervise all research activities so that all study steps and results could be harmonized. A health center-based clinic was set up for its central location in Xiangyang town. To obtain support from administrative authorities, the research group invited governmental representatives from Yueqing City and the Xiangyang Town.

### Primary outcome measures

1. Visual impairment: They were defined using the World Health Organization (WHO) 2003 criteria. No visual impairment: PDVA ≥ 6/12, mild impairment: 6/18 ≤ PDVA < 6/12, moderate visual impairment: 6/60 ≤ PDVA < 6/18, severe visual impairment 3/60 ≤ PDVA < 6/60, and blindness: PDVA < 3/60 in the better eye [72].
2. Refractive error and anisometropia: Myopia and hyperopia referred to a spherical equivalent (SE) ≤ − 0.5 D and ≥ + 0.5 D, respectively (also alternatively as ≤ − 1.0 D and ≥ + 1.0 D). Astigmatism was defined as minus cylinders ≤ − 0.5 D or ≤ − 1.0 D. Anisometropia was defined as a SE difference between the right and left eyes based on 1.0 D or 2.0 D [73].
3. Pterygium: The diagnosis of pterygium was made with a slit-lamp microscope and was defined as conjunctival tissue growth onto the clear cornea without an alternative explanation (for example, trauma). Pterygium was graded in 3 levels of severity based on relative transparency of pterygium tissue as follows: grade 1 (transparent), grade 2 (intermediate) and grade 3 (opaque) [74].
4. Cataract: The LOCS II [65] was used to assess the severity of lens opacity under the slit lamp examination in three major characteristics: cortical (C), nuclear opalescence (N), posterior subcapsular (P). Any cataract surgery was recorded with a history of cataract surgery in at least one eye.
5. Glaucoma suspect [75]: A glaucoma suspect was considered if any of the following characteristics were observed in either eye:
  1. IOP > 21 mmHg;
  2. Optic disc margin hemorrhage on the fundus disc-centered photography;
  3. Cup-disc ratio (CDR) ≥ 0.65, CDR asymmetry ≥ 0.2 or neural rim tissue ≤ 0.1;
  4. Diffused or localized retinal nerve fiber layer defect on fundus photography.

Glaucoma was confirmed by a reliable visual field defect with corresponding structural damage in the suspect’s eye [76].

6. Diabetic retinopathy and macular edema (ME): Fundus images were assessed with a masked mode according to the grading criteria applied in the Multi-Ethnic Study of Atherosclerosis (MESA), which was modified based on the Airlie House Classification system [77]. If one eye was unavailable for classification, the other one was graded. The severity of DR depended on the worse eye and each eye was assessed as follows: no DR (levels 10–13) or any DR (levels 14–80). DR was further classified as minimal non-proliferative diabetic retinopathy (NPDR) (levels 14–20), mild-moderate NPDR (levels 31–41), and severe NPDR to proliferative retinopathy (levels 51–80).
  Macular edema (ME) was identified when hard exudates co-occurred with blot hemorrhage and microaneurysms within 1 disc-diameter from the fovea or focal photocoagulation scars left in the macula. Clinically significant macular edema (CSME) was further confirmed based on the presence of ME within 500 μm from the central fovea or focal photocoagulation scars in the macula. Vision-threatening diabetic retinopathy (VTDR) was defined as the presence of CSME, severe NPDR, or PDR.
7. Age-related macular degeneration: AMD was graded using the Wisconsin AMD classification system [2].

### Statistical analysis and quality control

Statistical analyses were performed with standard statistical software (SPSS V24). Prevalence evaluation for diagnosed outcomes was determined in gender and age-stratified subsets. Gender and the age-adjusted prevalence rate were estimated according to the Chinese population from the 2010 China census [78]. For normally distributed data, an independent *t*-test or one-way analysis of variance (ANOVA) was used to compare the differences between groups. A Mann-Whitney U test or Kruskal-Wallis test was used for non-normal data. The Chi-square test was employed to analyze the different prevalence with respect to age- and gender-groups. Differences in multiple testing were adjusted with Bonferroni correction to control for the false discovery rate. Binary logistic regression was performed to identify the demographic variables associated with response to participate and risk factors for ocular disease.

The factors related to ocular diseases would be analyzed with single factor regression analysis. The significant different factors would be put into multiple regression analyses. We planned to build three types of risk prediction models: 1) The common risk factors-logistic risk prediction model; 2) Metabolomic markers-risk prediction model; 3) Combined model of two previous types. The area under the curve would be calculated using the receiver operating characteristic curve (ROC). We also use DeLong’s test to compare the performance of model prediction based on the ROC. Nomogram plot would be built on R software and bootstrap resampling will be used for internal validation.

While implementing the study, we undertook the quality control processes simultaneously. The ophthalmologists, clinicians, and assistant staff were trained to understand the purposes of the study, the diagnostic criteria, and standardized examination procedures. Eighty subjects for a pilot study were examined to ensure data consistency by verifying the repeatability of the examination and diagnosis results by paired examiners. An experienced ophthalmologist (LW) made the final decision about the different opinions from two graders. Investigators stayed at the field site to conduct and monitor the standardized procedures. Data were collected with a combination of paper and electronic edition. Paper data were input by double entry and validation.

## Results

4769 (89.7% participation rate) out of 5319 eligible residents participated in the physical and ocular examination. All subjects were self-reported to be from the Han-race population. Of the 4769 subjects, 3206 (67.2%) were examined in the health center, 1534 (31.7%) at the village clinic, and 52 (1.1%) at home (Fig. 2). Among the non-participants, 34 residents (6.2%) refused to participate in the examination, 157 residents (28.5%) agreed to home visits but were not present after 3 appointments, and 359 residents (65.3%) were temporarily out of Wenzhou City. Blood sample collection reached a sample size of 1909 (479 from diabetes subjects and 1430 from the remaining one-third of 4230 subjects).

Table 2 compares the demographic characteristics between the participants and non-participants. Most of the participants were females (54.4%). The median age of participants was 62.0 years (range from 50 years to 103 years), and the interquartile range was calculated (range from 56 years to 74 years). 75.3% had primary school education and below. The medical history and demographics are summarized in Table 2. In terms of co-morbidities history, 50.1% of subjects had a history of hypertension, 10.0% had diabetes, 2.2% had heart disease, and 1.1% had a history of stroke. Table 3 presents the outcomes of the anthropometric examination and biochemistry tests by gender for the participants.

**Table 2:** Comparing the characteristics of the participants with nonparticipants in the Yueqing Ocular Diseases Investigation

Characteristics	Participants		Non-participants		*P* value†
	Number	Percentage	Number	Percentage	
Gender					<0.001
Male	2175	45.6%	393	71.5%	
Female	2594	54.4%	157	28.5%	
Age (yrs)					<0.001
50–59	1884	39.5%	151	27.5%	
60–69	1375	28.8%	203	36.9%	
70–79	753	15.8%	113	20.5%	
80–89	664	13.9%	55	10.0%	
≥ 90	93	2.0%	28	5.1%	
Median age (P25, P75)	62 (56, 74)		66 (58, 73)		*P* = 0.001
Marital status					*P* = 0.154
Married	4026	84.4%	468	85.1%	
Unmarried	20	0.4%	4	0.7%	
Widowed	709	14.9%	74	13.5%	
Divorced	14	0.3%	4	0.7%	
Educational background					<0.001
Illiteracy or semi-illiteracy‡	1419	29.8%	106	19.3%	
Primary school (1–5 years)	2172	45.5%	259	47.1%	
Middle school (6–8 years)	958	20.1%	139	25.3%	
High school or above (9 years or above)	156	3.3%	36	6.5%	
Unknown	64	1.3%	10	1.8%	
History of diseases					
History of hypertension	2390	50.1%			
History of diabetes	479	10.0%			
History of heart diseases	107	2.2%			
History of stroke	51	1.1%			
Sample collection					
Serum and Plasma	1909	40.0%			

†Chi-square test for categorical variables: gender, age groups, marital status, and educational background; Mann-Whitney U test for abnormal variables: age; and binary logistic regression analysis for the association of gender (*P* < 0.001), age (*P* = 0.004), educational background (*P* = 0.013) with a response to participate

‡“illiteracy” was defined as an inability to read any Chinese words; “semi-illiteracy” was defined as having some understanding of Chinese words, but obtained little to no useful information through reading

**Table 3:** Outcomes of anthropometric examination and biochemistry tests in the Yueqing Ocular Diseases Investigation

	Male (n* = 2175)	Female (n* = 2594)
Height (cm)	164.7 ± 6.4	154.1 ± 6.1
Weight (kg)	66.1 ± 10.0	58.3 ± 9.5
BMI (kg/m^2^)	24.3 ± 3.1	24.5 ± 3.6
Body Surface Area (m^2^)	1.8 ± 0.2	1.7 ± 0.2
Waist circumference (cm)	87.0 ± 8.9	84.0 ± 9.6
Hip circumference (cm)	92.4 ± 4.9	93.0 ± 5.8
Waist hip ratio	0.9 ± 0.1	0.9 ± 0.1
Mean SBP (mmHg)	136.4 ± 15.5	137.2 ± 16.4
Mean DBP (mmHg)	80.1 ± 9.1	79.4 ± 8.8
Heart rate (/minute)	64.0 ± 5.5	64.7 ± 5.4
FBG (mmol/L)	6.1 ± 1.9	6.1 ± 2.0
HbA1c (%)	5.9 ± 0.6	6.0 ± 0.6
TC (mmol/L)	5.2 ± 1.1	5.6 ± 1.1
TG (mmol/L)	1.5 (1.0, 2.1)	1.5 (1.1, 2.2)
LDL-C (mmol/L)	3.3 ± 0.9	3.6 ± 1.0
HDL-C (mmol/L)	1.4 (1.2, 1.7)	1.5 (1.3, 1.8)
Scr (μmol/L)	79.7 (70.3, 89.8)	64.1 (57.2, 75)
BUN (mmol/L)	5.4 (4.3, 6.5)	4.9 (4.0, 6.1)

*“n” refers= to the number of examined male or female subjects

Normally distributed continuous variables are expressed as the mean ± standard deviation. Non-normal data are shown as the median (interquartile range)

*Abbreviations*: *BMI=* body mass index, *SBP=* systolic blood pressure, *DBP*=diastolic blood pressure, *FBG=* fasting blood glaucoma, *HbA1c=* glycosylated hemoglobin, *TC=* total cholesterol, *TG=* total triglycerides, *LDL-C=* low density lipoprotein-cholesterol, *HDL-C=* high density lipoprotein-cholesterol, *Scr=* serum creatinine, *BUN=* blood urea nitrogen

The primary prevalence rate of visual impairment among three examination sites was significantly different (Table 4). The prevalence of moderate visual impairment and worse was 9.8% (462/4697) in total; 35.7% (15/42) subjects were examined at home, 12.4% (182/1473) at the village clinics, and 8.3% (265/3182) at the health center.

**Table 4:** Prevalence rate of visual impairment in three examination sites

Level of visual impairment	n^*^	Home	Village	Health center	*P* value†
Blindness (PDVA < 3/60)	74 (1.6%)	12/42 (28.6%)	35/1473 (2.4%)	27/3182 (0.8%)	
Severe visual impairment (3/60 ≤ PDVA < 6/60)	34 (0.7%)	2/42 (4.8%)	12/1473 (0.8%)	20/3182 (0.6%)	
Moderate visual impairment (6/60 ≤ PDVA < 6/18)	354 (7.5%)	1/42 (2.4%)	135/1473 (9.2%)	218/3182 (6.9%)	
Mild visual impairment (6/18 ≤ PDVA < 6/12)	772 (16.4%)	3/42 (7.1%)	314/1473 (21.3%)	455/3182 (14.3%)	
No visual impairment (PDVA ≥ 6/12)	3463 (73.7%)	24/42 (57.1%)	977/1473 (66.3%)	2462/3182 (77.4%)	
Total‡	4697	42/4697 (0.9%)	1473/4697 (31.4%)	3182/4697 (67.7%)	< 0.001

*“n” refers to the total numbers of different degrees of visual impairment from the three examination sites

†Kruskal Wallis test

‡PDVA was collected from 4697 persons

*PDVA*= presenting distance visual acuity

## Discussion

The YODI aims to offer population-based information concerning the prevalence and risk factors of common visual impairing ocular diseases in a developed county population. This population was subsequently followed up and compared with another population in Wenzhou for external validation of metabolomics results. Data from this study will also provide early indicators for high-risk groups based on metabolomics characteristics of visual impairing diseases for developed areas in eastern China.

There are some important features of the YODI. First, we performed a clustered randomized sampling to improve the representativeness of the population and increase the rate to response (89.7%). Second, we chose the same diagnostic criteria used in other studies worldwide as well as in the local area to obtain comparable and reliable results. For example, the definitions of glaucoma used in the YODI were also used in the Wenzhou Glaucoma Screening Program [75]. We also implemented the same protocol of sample collection as the other metabolomics study [43] for clarity and consistency. Third, we employed fluently bilingual staff to decrease language and cultural barriers since, many aged residents only speak the Yueqing local dialect. Finally, with government investment and strict regulations over the last decade (From 0 to 45 yuan per person each year [79]), primary healthcare services have covered over 70% of the population in China now. Self-reported medical illness could be more accurate and representative of the prevalence of disease than that from the previous study in 2008 [28].

Our current study dose have some limitations. First, 3206 subjects (67.2%) were examined completely at the central clinic. The remaining subjects (32.8%) were performed with a non-corrective examination in the village clinic or at home in order to improve the participation rate. For consistency, we used the latest unified WHO (2003) definitions replacing BCVA with PDVA for visual impairment. However, the definitions could overestimate the prevalence of visual impairment disease resulting from ametropia [80, 81]. Second, most nonparticipants were temporarily working outside the City (6.7%, 359 of 5319 target population), because they were young and healthy with normal vision according to the previous health records. This selective bias could have caused an overestimation of visual impairment in the young group. Third, we reached 40% (1909/4769) coverage rate of sample collection in order to balance the confined storage space and samples to be used for multiple experiments. We planned to pick 100–200 samples in total out of the biobank, using propensity score matching of demographic data and laboratory tests for single eye disease group and normal control group to reduce data bias and confounding variables. Finally, we introduced LOCS-II in consideration of appropriate and enough grouped data in metabolic research, though it has higher tolerance limits than LOCS-III [82].

## Conclusions

In summary, the YODI provides population-based information with a high response rate (89.7%) on the prevalence and primary causes of major vision-impairing eye diseases in developed county areas in eastern China. Metabolism analysis from YODI will provide a further association of the metabolic characteristics with the visual impairment of eye diseases in China. The risk prediction model could be created and has the potential to be generalized to developed eastern areas in China for prevention.

## Data Availability

The datasets generated and analysed during the current study are not publicly available, because the local government urges there be no disclosure of any residents’ information. All relevant data supporting the findings of this study are available from the corresponding author upon request.

## Abbreviations

AMD: Age-related macular degeneration
ANOVA: Analysis of variance
BCVA: Best-corrected visual acuity
BUN: Blood urea nitrogen
CDR: Cup-disc ratio
CSME: Clinically significant macular edema
DBP: Diastolic blood pressure
DR: Diabetic retinopathy
EDTA: Ethylene diamine tetraacetic acid
FBG: Fasting blood glucose
GDS: Geriatric depression scale
HbA1c: Glycosylated hemoglobin
HDL-C: High density lipoprotein-cholesterol
IOP: Intraocular pressure
LDL-C: Low-density lipoprotein-cholesterol
LOCS: Lens opacities classification system
ME: Macular edema
MESA: Multi-ethnic study of atherosclerosis
MMSE: Mini-mental state examination
NPDR: Nonproliferative diabetic retinopathy
PDVA: Presenting distance visual acuity
ROC: Receiver operating characteristic curve
SBP: Systolic blood pressure
Scr: Serum creatinine
TC: Total triglycerides
TG: Total cholestero
VA: Visual Acuity
VTDR: Vision-threatening diabetic retinopathy
WHO: World health organization
YODI: Yueqing Ocular Diseases Investigation
